# Investigation of the Effect of High Shear Stress on Mesenchymal Stem Cells Using a Rotational Rheometer in a Small-Angle Cone–Plate Configuration

**DOI:** 10.3390/bioengineering11101011

**Published:** 2024-10-11

**Authors:** Mario Mand, Olga Hahn, Juliane Meyer, Kirsten Peters, Hermann Seitz

**Affiliations:** 1 Chair of Microfluidics, Faculty of Mechanical Engineering and Marine Technology, University of Rostock, 18059 Rostock, Germany; 2 Institute of Cell Biology, Rostock University Medical Center, 18057 Rostock, Germany; olga.hahn@med.uni-rostock.de (O.H.); kirsten.peters@med.uni-rostock.de (K.P.); 3 Human Med AG, 19061 Schwerin, Germany; juliane.meyer@humanmed.com; 4 Department of Life, Light and Matter, University of Rostock, 18059 Rostock, Germany

**Keywords:** rotational rheometer, cone and plate configuration, thin-gap rheometry, low viscous liquids, high shear rates, mesenchymal stem/stromal cells

## Abstract

Within the healthy human body, cells reside within the physiological environment of a tissue compound. Here, they are subject to constant low levels of mechanical stress that can influence the growth and differentiation of the cells. The liposuction of adipose tissue and the subsequent isolation of mesenchymal stem/stromal cells (MSCs), for example, are procedures that induce a high level of mechanical shear stress. As MSCs play a central role in tissue regeneration by migrating into regenerating areas and driving regeneration through proliferation and tissue-specific differentiation, they are increasingly used in therapeutic applications. Consequently, there is a strong interest in investigating the effects of shear stress on MSCs. In this study, we present a set-up for applying high shear rates to cells based on a rotational rheometer with a small-angle cone–plate configuration. This set-up was used to investigate the effect of various shear stresses on human adipose-derived MSCs in suspension. The results of the study show that the viability of the cells remained unaffected up to 18.38 Pa for an exposure time of 5 min. However, it was observed that intense shear stress damaged the cells, with longer treatment durations increasing the percentage of cell debris.

## 1. Introduction

Adipose tissue and adipose tissue-derived cells have been studied and used for aesthetic and regenerative applications for more than 20 years [1,2,3]. Adipose tissue in its entirety can be used, for example, for the treatment of scars and chronic wounds [4,5]. The regenerative properties of adipose tissue are not derived from the adipocytes themselves, but rather from the cells of the so-called stromal vascular fraction (SVF) contained therein [6]. The SVF also includes mesenchymal stem/stromal cells [1]. Due to their broad spectrum of differentiation and immunomodulatory capacities, adipose-derived mesenchymal stem cells (AD-MSCs) hold great therapeutic promise, as evidenced by their extensive involvement in clinical trials [7,8,9,10,11,12,13,14]. In order to ensure the most efficient use of the cells, it is necessary to isolate the SVF from the adipose tissue. This can be achieved through different methods. The most widely established method is the enzymatic digestion of the tissue with collagenase to dissociate the connected cells into a single-cell suspension [15]. While this is the most effective way to separate the SVF, enzymatic digestion is regarded as a substantial tissue manipulation by the European Medicines Agency, which makes the enzymatically generated SVF an advanced medical therapy product (ATMP) [16] that would need to be manufactured under the EU Guidelines for Good Manufacturing Practice (GMP) conditions. These conditions are not easily met in an operating theater setting. Thus, the non-enzymatic isolation of SVF from adipose tissue, which is not considered a substantial manipulation, has gained popularity in the medical field. All non-enzymatic methods utilize mechanical force in order to selectively destroy the adipocytes that are present and separate the stromal cells from the tissue compound [17,18,19]. Several shearing-based dissociation methods have been investigated over recent years [20,21,22,23,24]. One aspect, which has not been investigated conclusively, is the influence of the high shear stress that is exerted upon the AD-MSCs during these procedures.

Shear stress is widely recognized for its significant and far-reaching impact on cellular behavior and function. For example, the influence of shear stress on blood cells is an extensively researched topic, because the components of blood are constantly exposed to fluid shear stress (FSS) [25]. It has been shown that not only the intensity of shear stress but also the time of exposure has an impact to the cells.

Various methods have been developed to apply shear stress to cell suspensions. Approaches such as the Couette shearing device are feasible for the high-throughput shearing of a cell-loaded fluid [26,27]. In recent years, microfluidic devices have been increasingly employed to induce shear stress in single cell solutions. These microfluidic devices and cannulas are generating FSS due to the flow through a narrow cavity [28,29,30,31].

A common device for generating defined shear stress in cells is a rotational rheometer. Rotational rheometers are employed to investigate the influence of FSS on, e.g., the activation of T cells or to test the resistance of cancer cells to FSS [32,33,34,35] and to investigate the effect on platelet activation [36,37] and the induction of shear stress to blood for a rheological study of blood behavior at different shear rates [38,39,40]. While the so-called double-wall geometry is the preferred geometry for standard shear rheometry, some studies use cone–plate geometry to take advantage of the uniform shear rate distribution in the sample volume [39,40,41,42,43]. A physiologically relevant shear stress can be easily achieved with conventional rheometric geometries. However, while attempting to achieve a higher shear stress by increasing shear rates, the flow pattern in the sample volume tends to diverge from the desired tangential flow to a more complex behavior, making it more difficult to predict the actual shear force to the cells in suspension. An approach to counteract this transition is to use a plate–plate geometry with a narrow gap. The narrow gap (10–100 µm) enables the attaining of higher shear rates while ensuring a tangential laminar flow in the sample volume. The downside of the plate–plate geometry is a non-uniform shear rate distribution in the sample volume following an uneven shear force induction to the cell sample [44,45]. Secondly, plate–plate geometry approaches are commonly conducted on a cell monolayer adhered to a flat surface inserted into the rotational rheometer, though this needs additional preparation time.

In conclusion, the plate–plate geometry is not the most suitable option for applying high shear rates to single-cell suspensions to investigate the impact of high shear stress on cells. Unlike the plate–plate geometry, the cone–plate geometry has a uniform shear rate distribution over the whole sample volume, inducing consistent shear stress to the cell sample [46]. Due to its uniform shear rate distribution, a cone–plate geometry would be more suitable for shear stress studies [47], but so far, no studies have been found that perform high shear rate experiments with single-cell solutions of native biological tissue using a cone–plate geometry.

In this study, we present a set-up for applying high shear rates to cells based on a rotational rheometer with a small-angle cone–plate configuration. The cone used features a small angle, generating a narrow gap between the cone and plate geometries, enabling the attainment of high shear rates. This set-up was used in an initial study to investigate the effect of high shear stress on cultured AD-MSCs in suspension, with particular emphasis on viability and proliferation depending on shear stress and exposure time.

## 2. Materials and Methods

### 2.1. Concept

In order to establish a method for applying high shear rates to cells, it was necessary to establish an experimental protocol. For this purpose, a sample of cultured, non-adhered human AD-MSCs in a suspension of a low-viscosity medium served as the test sample. As the shear stress is not applied directly to the cells, but via fluid friction, it had to be ensured that the carrier fluid had constant and reproducible rheological properties over a suitable shear rate range [48,49].

### 2.2. Carrier Fluid

A cell culture medium was intended to serve as a carrier fluid for the shear experiments, as this meant that no further cell-processing steps needed to be carried out with regard to the shear experiments. In order to identify a suitable carrier fluid and to be able to quantify the shear stress exerted on the cells, the rheological properties and the density of commonly used standard cell culture media had to be analyzed [15]. On the one hand, the carrier fluid should exhibit a density as similar as possible to that of the cells to be examined, and, on the other hand, it should have constant rheological properties over time. The rheological behavior of the carrier fluid can be influenced by the different components of the cell culture medium mixture. Therefore, the components were initially investigated in different permutations of the ingredients to evaluate the most suitable mixture.

The first variant and main component of the culture medium was Dulbecco’s Modified Eagle’s Medium with high glucose content (Gibco by Life Technologies, Darmstadt, Germany, referred to as DMEM), while in the second approach both 0.4% GlutaMax™ and 1% penicillin/streptomycin (penicillin: 100 U/mL, streptomycin: 100 mg/mL, both Gibco by Life technologies, Darmstadt, Germany) were added (referred to as DMEM+G). The third approach examined was the established mixture for cell culture cultivation, which contained 10% of fetal calf serum (referred to as DMEM+G+FCS). The geometry and methodology employed in this study were thoroughly validated using ultrapure water filtered by the ELGA Purelab Flex 1 (Veolia Water Technologies Deutschland GmbH, Celle, Germany).

### 2.3. Media Density

In order to minimize both radial migration due to rotational forces acting on the cells suspended in the medium and sedimentation in the shearing process, it is essential to use a carrier fluid with a density that corresponds to the density of the cells. The measurement instrument employed for this purpose was the DSA-5000-M (AntonPaar GmbH, Graz, Austria). The density of three possible carrier fluids, DMEM, DMEM+G and DMEM+G+FCS, was determined and compared to the cell density evaluated by Drobek et al. [50]. All density measurements of the media were conducted at a controlled temperature of 25 °C, as this temperature was consistent with all measurements performed.

### 2.4. Rheometer Test Set-Up

**Figure 1:**
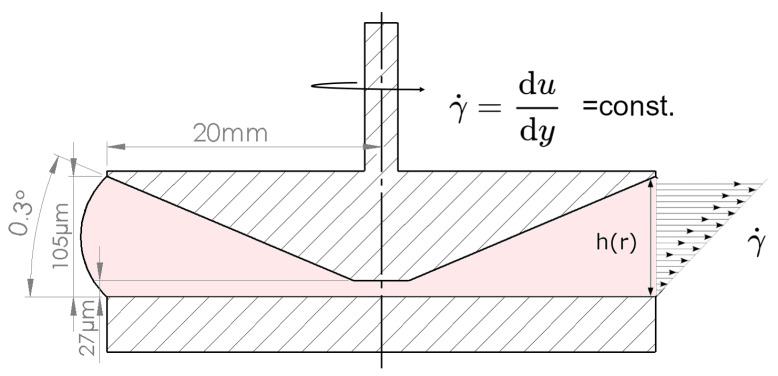
Schematic illustration of the cone–plate geometry. As the size of the gap increases with the radius, the shear rate $\dot{\gamma }\left(r\right)$ is uniform in the measurement geometry in laminar flow conditions. Geometries are not shown to scale.

The rheometer used for these experiments was the Anton Paar MCR 702 Multidrive, (AntonPaar GmbH, Graz, Austria). The measurement geometry was a cone–plate geometry, and it consisted of a small-angle CP40-0.3 cone (d: 40 mm, α: 0.3°, see Figure 1) and an I-PP40/SS plate (d: 40 mm). Temperature control was achieved using the P-PTD200/62/TG Peltier temperature control device, which operates with water heating. All measurements were conducted at a controlled temperature of 25 °C ± 0.1 °C. Only constant shear rate procedures were recorded, with the rotational speed adjusted to match the desired shear rate with an accuracy of ±0.1%. Before every measurement, the cone–plate geometry was cleaned using 70% ethanol, followed by wiping with 500 µL of ultrapure water. The dryness of the surfaces was achieved using a lint-free paper towel. Any remaining particles were eliminated using compressed air. The cleaning procedure was performed before every measurement.

The cone–plate geometry was chosen as it enables a uniform shear rate distribution over the entire measurement volume. Here, the shear rate remains uniform, if the laminar flow conditions are met. In this scenario, the centrifugal forces can be considered negligible compared to the viscous forces within the fluid [38]. The fluid shear stress on a flowing cell in laminar flow is defined as

(1) $$\tau =\eta *\dot{\gamma }$$

where *τ* is the shear stress, *η* the dynamic viscosity of the medium and $\dot{\gamma }$ the shear rate. The shear rate $\dot{\gamma }$ in a rotating cone–plate geometry is calculated as follows:

(2) $$\dot{\gamma }\left(r\right)=\frac{r*\omega }{r*\tan\left(\alpha \right)}=\dot{\gamma }$$

where r is the radius, ω the angular velocity and α the cone angle. Due to the angle of the cone, a uniform shear rate is achieved, ensuring a uniform shear stress over the measuring volume and thus on all sample cells [46,51]. In the sample fluid in rotating cone–plate geometries, two transitions in the flow field occur: from a fully tangential laminar flow to a laminar flow with secondary flow behavior and to a completely turbulent flow at a specified critical Reynolds numbers Re∗ [48]:

(3) Re∗=γ˙r2α3ρη,

Ellenberger and Fortuin found that for ${Re}_{tang}^{*}\leq 1,$ the fluid flow is fully tangentially laminar. In shear rates over $Re_{tang}^{*}>1$, secondary flow patterns occur. The emergence of secondary flow patterns results in an elevation in the measured torque, consequently leading to an augmentation in the measured viscosity. A correction factor for the actual shear stress can be determined using an equation for the relative torque [48].

Sgoudos et al. [49] found that at $Re_{sec}^{*}\geq 6$, the flow contains secondary flow components that exert a considerable torque on the rotating cone. Also, the threshold value for the transition from laminar to turbulent flow in a cone–plate geometry is $Re_{turb}^{*}=48$. The secondary flows intensify with an increase in shear rate until a laminar–turbulent transition occurs, at which point the flow becomes completely chaotic. The fully tangential laminar flow is guaranteed up to a shear rate of [48]

(4) $$Re_{tang}^{*}=1\;:\;{\dot{\gamma }}_{tang}^{*}=1.55*10^{4}{\;s}^{-1}\;,$$

Meanwhile, secondary flows that exert a considerable torque would occur at a shear rate of [49]

(5) $$Re_{sec}^{*}=6\;:\;{\dot{\gamma }}_{sec}^{*}=9.32*10^{4}{\;s}^{-1}\;,$$

following Equation (3) for water at 25 °C.

To test the suitability of the different cell culture media as a carrier fluid, the rheological behavior of carrier fluids has to be examined across a wide range of shear rates and exposure times. The results also allow the shear stress on cells to be quantified.

### 2.5. Influence of Exposure Time

Our experimental design included an investigation of sheared cell carrier media over periods of 5 and 10 min, with constant shear rates applied to the fluid. This was undertaken to evaluate the time-dependent behavior of the cell carrier medium, considering factors such as the possible agglomeration of dissolved contents within the carrier fluid and the potential for evaporation from the measuring volume. During the experiments, a logarithmic ramp was applied, transitioning from an initial shear rate of γ = 500 s^−1^ to a constant shear rate in the range of γ = 1.0 × 10^4^ s^−1^–3 × 10^4^ s^−1^. The constant shear rate was maintained over a period of 10 min.

### 2.6. Influence of Shear Rate

The objective of this part of the study was to investigate the dynamic viscosity of cell carrier media at high shear rates. In particular, we sought to observe potential shifts in viscosity with increasing shear rates and to examine the potential limitations associated with high shear rates, considering various fluid-mechanical phenomena. To achieve these goals, we conducted an evaluation of dynamic viscosity over a wide range of shear rates. Torque measurements were taken at constant rotational speeds, corresponding to shear rates in the range of γ = 5.0 × 10^2^ s^−1^–5 × 10^4^ s^−1^. The targeted range exceeds the shear rate for laminar tangential flow, yet the shear rate remained below the threshold for strong secondary flows. The shear rate was increased logarithmically in steps to obtain 20 measurement points per decade. As a result of the logarithmic increase in shear rate and averaging process, the measurement time also followed a logarithmic pattern. This resulted in measurement times from 5 s at γ = 5.0 × 10^2^ s^−1^ to 0.5 s at 5 × 10^4^ s^−1^, giving a total measuring time of 81 s for each measurement. Due to the limited time window, which is primarily due to the consideration of time dependency effects, we did not include a ramp-down phase.

### 2.7. Preparation of Cell Suspension

To evaluate the impact of shear stress on the cells, we used the cell type of primary human AD-MSCs as an example. These AD-MSCs were obtained from 6 healthy patients (female with a mean age of 49.17 ± 14.16 years and a body mass index of 24.01 ± 2.18 kg/m^2^) who underwent water jet-assisted liposuction. The isolation of these cells was performed according to the previously described protocol [52,53,54]. For all cell culture experiments, AD-MSCs from passage 2 were thawed in DMEM with 10% FCS, 1% penicillin/streptomycin and 0.4% GlutaMAX™ according to a standard laboratory protocol and seeded in a 75 cm^2^ flask (Greiner Bio-one, Frickenhausen, Germany) [54] for 5–6 days to reach approx. 90% confluence. For all shear experiments, AD-MSCs in passage 3 were used. For this purpose, cells were washed twice with PBS without Ca^2+^ and Mg^2+^ (PAN Biotech, Aidenbach, Germany) and detached from the cell culture flaks by incubation with 0.25% Trypsin-EDTA (Gibco by Life technologies, Paisely, UK) for 5 min at 37 °C in a humidified atmosphere. Detachment was confirmed microscopically and stopped with DMEM containing 10% FCS, 1% penicillin/streptomycin and 0.4% GlutaMAX™. Since all experiments were conducted with DMEM without FCS, the cell suspension was then centrifuged at 400× *g* for 5 min at RT. The resulting cell pellet was resuspended in 4.2 mL DMEM containing 1% penicillin/streptomycin and 0.4% GlutaMAX™ (DMEM+G), and the cell count was performed using the Nucleocounter^®^ NC-200™ (Chemometec, Lillerod, Denmark) “viability and cell count assay” according to the manufacturer’s instructions, with a two-fold determination. The absence of mycoplasma contamination in the AD-MSCs was confirmed microscopically by DNA staining with DAPI (Sigma Aldrich, Saint Louis, MO, USA). Unless otherwise stated, all plastic wares were from Greiner Bio-One (Frickhausen, Germany).

### 2.8. Fluid Shear Stress to AD-MSC

In order to evaluate cellular responses to mechanical forces, shear stress experiments were carried out with AD-MSCs. For this purpose, the cleaning process described above was performed before each experiment. For each shear experiment, 250,000 cells per 110 µL of carrier fluid were prepared and added to the geometry as a droplet. The average cell diameter of 20 µm and the assumption that the cells are perfect spheres were used to calculate the volume fraction of 0.98% [50]. The cell suspension was then sheared at shear rates of γ = 0.5 × 10^4^ s^−1^–3 × 10^4^ s^−1^ for 5 or 10 min. For this purpose, a consistent ramp time of 8 s was maintained for the acceleration in all experiments, which consequently led to different accelerations. The torque was measured continuously during shearing to assess the divergent forces at 1 Hz caused by the presence of cells in the medium. In addition, cell suspensions without rotation in the rheometer were used as control measurements. After the shearing process, 50 µL of the cell suspension was removed from the test system and filled up with 500 µL DMEM+G+FCS for cell counting in a Nucleocounter^®^ NC-200™ (viability and cell count assay) and further cultivation. For the subsequent cultivation, the cells were seeded in a 6-well plate and cultivated in DMEM+G FCS for a period of up to 12 days.

### 2.9. Evaluation of Further Cell Cultivation and Morphological Changes

Due to the experimental set-up, the experiments were carried out in a non-sterile manner, so that microscopic documentation was first carried out to exclude any possible contamination. Possible morphological changes in the organization of the cytoskeletal structure were visualized with the help of F-actin staining. For this purpose, the cell cultures were washed twice with PBS containing Ca^2+^ and Mg^2+^ (Cytiva Hyclone™ Dulbecco’s Phosphate-Buffered Saline, Fisher Scientific, Hampton, New Hampshire, USA) for 2 min each and fixed with 4% paraformaldehyde at RT for 10 min, followed by two 2 min wash steps with PBS without Ca^2+^ and Mg^2+^. Cells were then permeabilized with 0.1% Tween in PBS without Ca^2+^ and Mg^2+^ for 5 min, blocked with 1% bovine serum albumin in PBS without Ca^2+^ and Mg^2+^ for 2 min and incubated with Alexa Fluor^®^ 594 phalloidin (6.6 µM with emission: 609 nm and excitation: 581 nm) for 20 min in the dark. Before embedding the cells with Fluoroshield with DAPI (ImmunoBioSciences, Mukilteo, Washington, USA) with emission: 360 nm and excitation: 460 nm, and the cells were washed twice with PBS without Ca^2+^ and Mg^2+^. Images were taken with an Observer Z1 fluorescence microscope, version1.2 (Carl Zeiss Microscopy Deutschland GmbH, Oberkochen, Germany).

### 2.10. Data and Statistics

The media density measurements were conducted three times for statistical reasons. Furthermore, each exposure time experiment was repeated three times, and the arithmetic mean was calculated for each measurement point. The measurement was conducted at 2 Hz, leading to a total of 1200 recorded measured values at 10 min exposure time (resp. 600 values at 5 min). To minimize measurement errors due to geometric inaccuracies, all torque measurements were averaged over a full rotation (phase average). The shear rate sweep measurement was performed six times, and the arithmetic mean was determined for each measuring point. The measurement trigger frequency was logarithmically increased to match the period length. Viscosity data acquisition was performed using *RheoCompass* (AntonPaar GmbH, Graz, Austria), data analysis was carried out using *MATLAB* (2021a, The MathWorks Inc., Natick, MA, USA), and graphical representations were created using *Origin* (2021, OriginLab Corporation, Northampton, MA, USA).

All biological experiments were performed independently with AD-MSCs from 6 donors in 2–3 technical replicates each, using the mean of each replicate for one individual. Data were visualized and statistically analyzed using Microsoft Excel 2010 and GraphPad Prism, version 7 (GraphPad Software Inc., San Diego, CA, USA). The data were presented as boxplots, with the horizontal line within the boxplot indicating the median and the mean (+) and the whiskers indicating the minimum and maximum values. Depending on the normal distribution of the data (Shapiro–Wilk test), statistical significance was calculated using an ordinary one-way Analysis of Variance (ANOVA) with Dunnett’s multiple comparison test or by Kruskal–Wallis with Dunn’s multiple comparison test, and the significance level was set at * *p* < 0.05 (significant compared to Ctrl.).

### 2.11. Ethics

All experiments were conducted after receiving full consent of the patients, with positive ethics approved by the local ethics committee (Rostock University Medical Center) under registration number A2013-0053.

## 3. Results

### 3.1. Media Density

**Figure 2:**
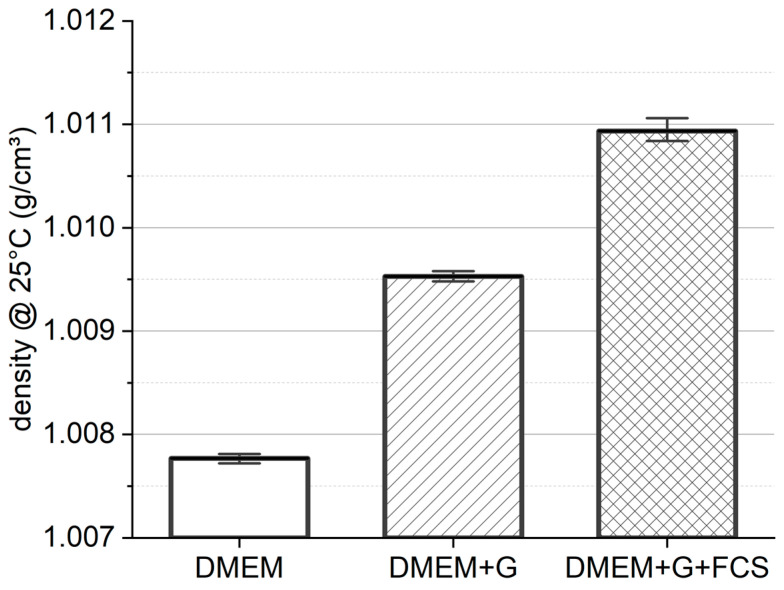
Media density. Density measurements were performed at 25 °C and ambient pressure (mean with standard deviation, n = 3).

To predict the behavior of cells in suspension during centrifugation and shear studies with regard to radial migration, we first measured the density of the carrier fluid. While DMEM has the lowest density with $\rho_{DMEM}$ = 1.0077 ± 0.4 $\frac{g}{{\mathrm{cm}}^{3}}$ (see Figure 2), the addition of GlutaMAX™ increases the fluid density by about 0.2% to $\rho_{DMEM+G}$ = 1.0095 ± 0.4 $\frac{g}{{\mathrm{cm}}^{3}}$. The highest density was detected in DMEM+G+FCS, with $\rho_{DMEM+G+FCS}$ = 1.0109 ± 0.9$\;\frac{g}{{\mathrm{cm}}^{3}}$. The addition of 10% FCS increases the density of the medium by 0.3%. The observed standard deviation between our measurements ranged from 0.004% to 0.009%.

### 3.2. Influence of Shear Rate

**Figure 3:**
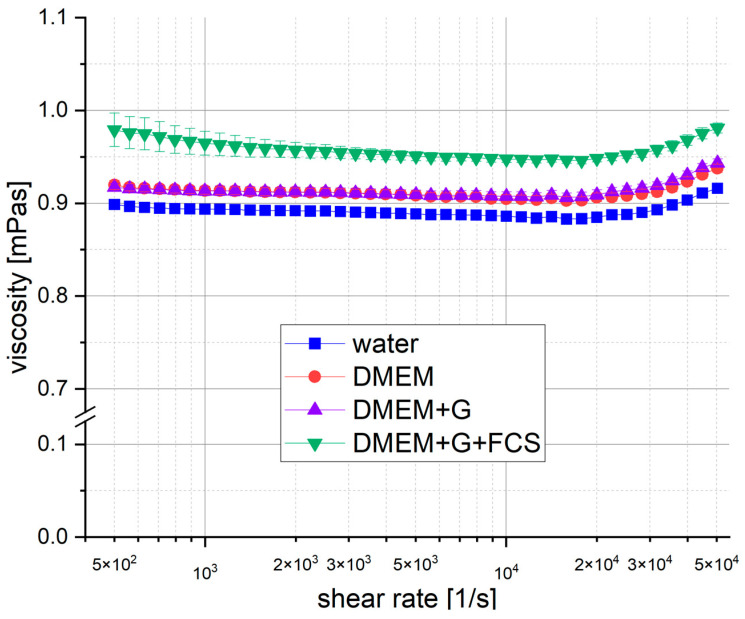
Influence of shear rate on dynamic viscosity of cell media. Viscosity measured for DMEM (red), DMEM+G (purple), DMEM+G+FCS (green) and water (blue) at 25 °C via rotational rheometer in small-angle cone–plate configuration (mean with standard deviation, n = 6).

In Figure 3, we present the data obtained from our measurements. At ${\dot{\gamma }}_{tang}^{*}$ = 1.55 × 10^4^ s^−1^, the apparent viscosity increased (see Section 2.4). This increase is due to secondary flow effects, resulting in an induced torque that exceeds the torque induced by viscous friction. The standard deviation between measurements remained remarkably low, with the exception of DMEM+G+FCS, which is particularly evident at low shear rates. The final constant values for the dynamic viscosity are found in Table 1.

### 3.3. Influence of Exposure Time

**Figure 4:**
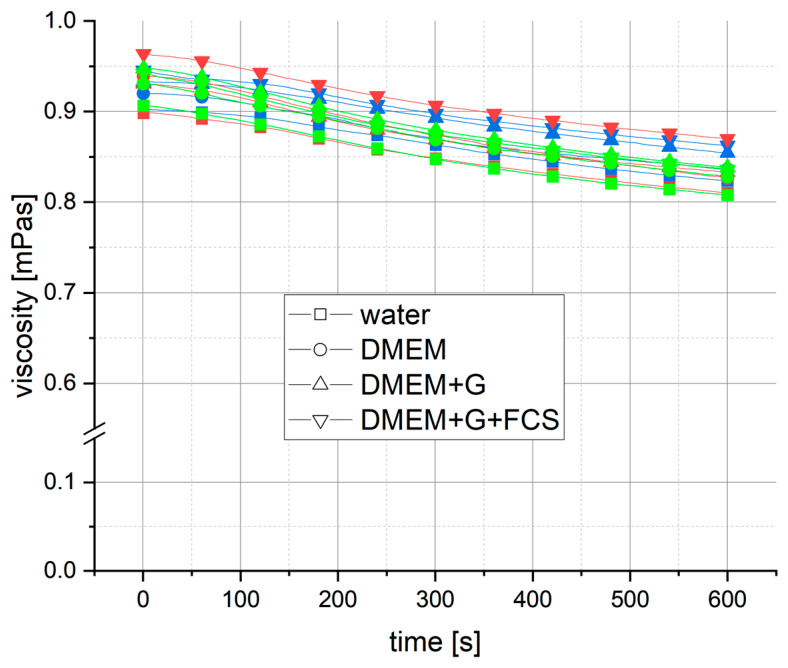
Viscosity behavior of cell media over exposure time of 10 min at constant shear rate of 1 (blue), 2 (red) and 3 × 10^4^ s^−1^ (green). Viscosity measured for DMEM (circle), DMEM+G (triangle up), DMEM+G+FCS (triangle down) and water (square) at 25 °C via rotational rheometer in small-angle cone–plate configuration. The decline in apparent viscosity over time showed no correlation with the shear rate and remained constant for all media and shear rates.

Initially, shear measurements were only performed with the media to investigate the behavior of the potential carrier fluids during the planned duration time. At a shear rate of γ = 1 × 10^4^ s^−1^, all media displayed a similar behavior, with an almost linear decrease in apparent viscosity over 10 min (Figure 4).

**Figure 5:**
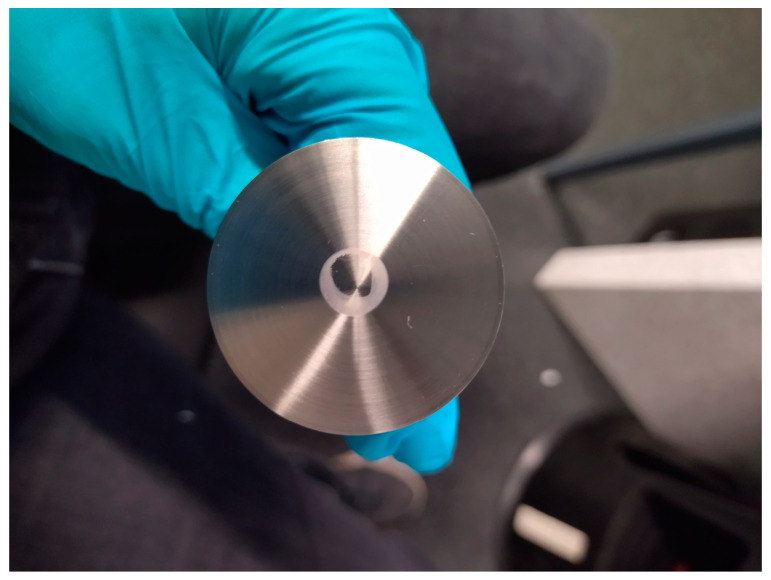
Agglomeration in center of the cone geometry during the rheological characterization of DMEM+G+FCS at 10 min exposure time. At shear rates of $\dot{\gamma }=3\times 10^{4}\;s^{-1}$, the proteins in the fetal calf serum appeared to agglomerate and adhered to the measurement geometry surface. The agglomeration led to the exclusion of DMEM+G+FCS as a possible carrier fluid.

The decrease in viscosity ranged from $\Delta \eta_{app}$ = −0.098 mPas to −0.109 mPas. The decrease in apparent viscosity initiated at a low rate, with the first minute exhibiting a reduction not exceeding $\Delta \eta_{app}$ = −0.004 mPas (except for DMEM+G+FCS). This decrease in apparent viscosity began at low shear rates, with a decrease of no more than $\Delta \eta_{app}$ = −0.004 mPas in the first minute (with the exception of DMEM+G+FCS). After 2 min, the rate of decrease remained nearly constant, at approx. 0.01 mPas per minute, resulting in a linear reduction of apparent viscosity over time in all media. Over a period of 5 min, a maximum decrease in apparent viscosity of 5.1% was measured, while a maximum decrease in apparent viscosity of 8.5% was measured after 10 min. At higher shear rates of γ = 1 × 10^4^ s^−1^ and γ = 3 × 10^4^ s^−1^, the same behavior was observed. However, DMEM+G+FCS showed agglomeration in the center of the lower plate of the measurement geometry after a measurement of γ = 3 × 10^4^ s^−1^ at 10 min (see Figure 5). In a second measurement of the same sample, the apparent viscosity increased abruptly, indicating agglomeration throughout the whole height of the geometry gap.

Based on these results, we decided to use DMEM+G as the carrier fluid for the shear stress experiments, firstly because its density was closer to the density of the cells of $\rho_{adMSC}$ = 1.0525$\;\frac{g}{{\mathrm{cm}}^{3}}$ described in literature [50], and secondly because DMEM+G+FCS led to agglomeration and therefore could not be used for the biological cell experiments (Figure 5). We also found that its viscosity was consistently higher in all measurements compared to measurements with DMEM+G medium without cells. This is in line with the expected outcome, as the addition of cells (250,000/110 µL) naturally thickens the solution, resulting in a higher apparent viscosity. In the course of our experiments, we observed a consistent decrease in apparent viscosity in all measurements. Remarkably, the rate of decrease in apparent viscosity was similar to that observed in the exposure shear stress measurements with pure cell media.

### 3.4. Shear Stress to Human AD-MSC

**Figure 6:**
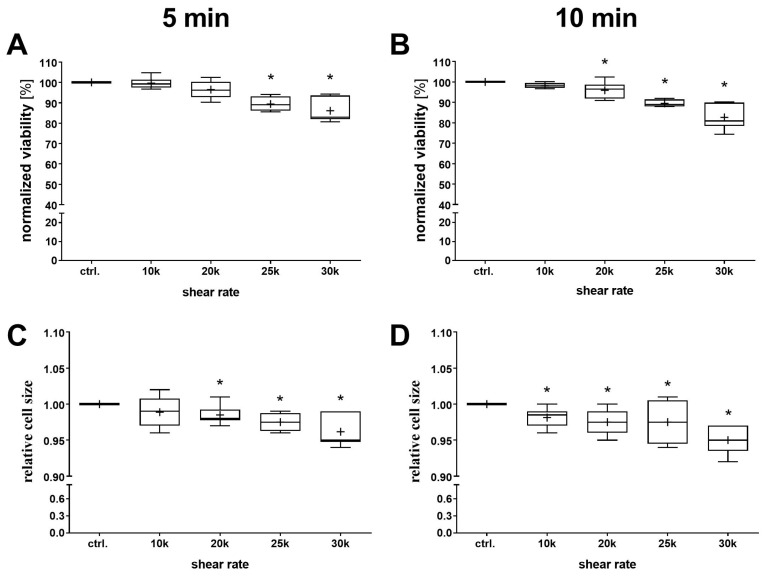
Characterization of viability and cell size after shearing. Quantification of cell viability (**A**,**B**) and relative cell size (**C**,**D**) after 5 min (**A**,**C**) or 10 min (**B**,**D**) shearing at different shear rates (1, 2, 2.5 and 3 × 10^4^ s^−1^). Cell cultures without starting the shearing procedure served as control (Ctrl.) cultures (the data set was normalized to the control, depending on the normal data distribution (Shapiro–Wilk test); statistical significance was calculated using an ordinary one-way ANOVA with Dunnett’s multiple comparison test or by Kruskal–Wallis with Dunn’s multiple comparison test (* *p* < 0.05 significant compared to the Ctrl., n = 6).

To determine to what extent the different shear rates affect AD-MSCs, viability and cell size after shearing was determined using the NC-200™ cytometer. A shear-dependent decrease in viability was detected for both exposure times (Figure 6A,B), with a significant decrease observed from γ = 2.5 × 10^4^ s^−1^ for the 5 min shear and already from γ = 2 × 10^4^ s^−1^ for the 10 min shear. This shear-dependent decrease in viability was simultaneously accompanied by a shear-dependent decrease in relative cell size (Figure 6C,D).

**Figure 7:**
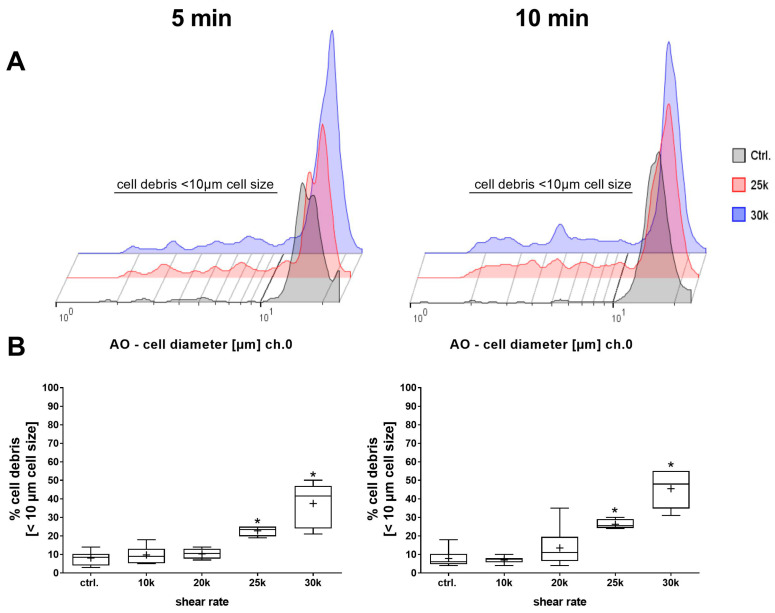
Analysis of the cell debris fraction after shearing. Representative histograms of cell diameter (**A**) and quantification of cell debris fraction, defined as cell sizes < 10 µm, (**B**) after 5 min or 10 min shearing with different shear rates (1, 2, 2.5 and 3 × 10^4^ s^−1^). Cell cultures without incipient shearing procedure served as control cultures (data set was normalized to control, Shapiro–Wilk test indicated non-normal data distribution, statistical significance was calculated using a Kruskal–Wallis test with Dunn’s multiple comparison test, * *p* < 0.05 significant compared to control cultures, n = 6).

A closer look at the cell size histograms (Figure 7A) shows that they exhibit additional deflections with an increasing shear rate. Subsequent analysis showed a significant shear-dependent increase in the cell debris fraction for both shear times, which, together with the relative cell size data, indicates a destruction of the AD-MSCs into smaller particles below 10 µm (Figure 7B).

**Figure 8:**
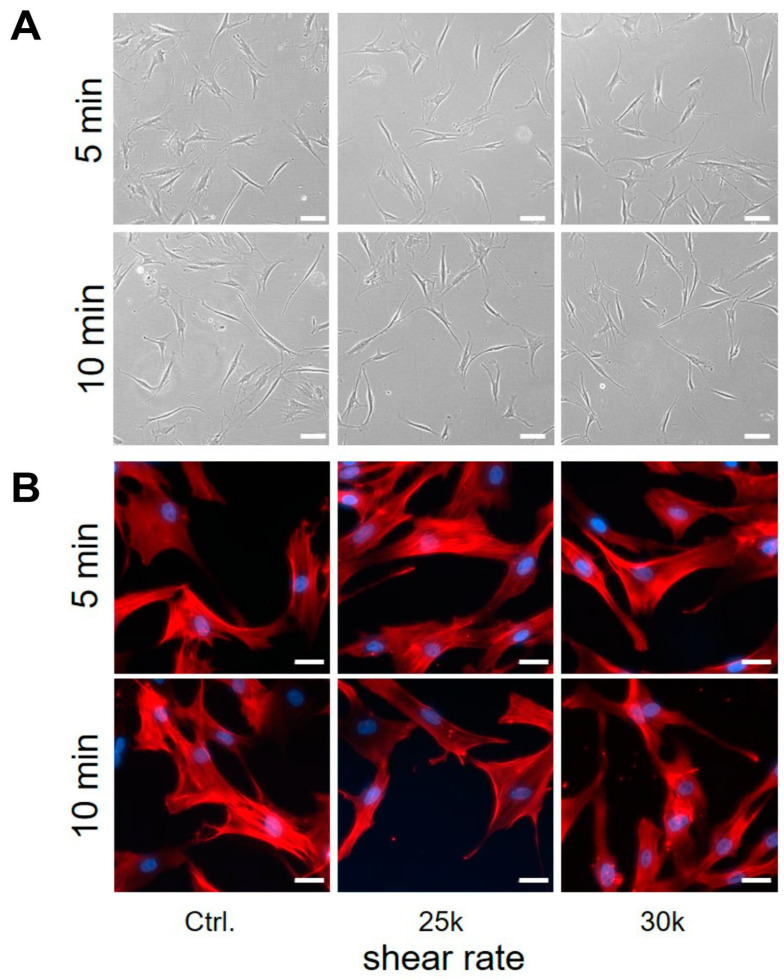
Effect of shear on adherence capacity and on the F-actin of AD-MSCs after 24 h. Representative images of adherent cell cultures (**A**) and F-actin (red) and nuclei (blue) staining (**B**) after 5 min or 10 min shearing with different shear rates (1, 2, 2.5 and 3 × 10^4^ s^−1^). Cell cultures without incipient shearing procedure served as control cultures.

To determine the influence of shearing on the ability of AD-MSCs to adhere to tissue culture plastic, the sheared cells were seeded in a 6-well plate for 24 h. After 24 h, phase microscopy images showed that AD-MSC adhered to the cell culture plates and could therefore be cultured further. For downstream analysis, the AD-MSCs were also detached and counted. Analysis of these data revealed no significant differences in the adherence capacity of AD-MSCs after shearing (Figure 8A). To assess the effects of shear on cell morphology, the F-actin of the AD-MSCs was stained (Figure 8B). The F-actin staining showed the characteristic appearance of actin filaments with slender linear structures in the cytoplasm in all cultures, indicating no differences in the cytoskeleton. As the shearing was performed in a non-sterile manner under the given circumstances, we investigated whether further cultivation of the cells was possible without signs of contamination. For this purpose, we cultivated the cells after shearing over a longer period of time. The microscopic documentation showed that cultivation was possible for up to 12 days without any signs of contamination.

## 4. Discussion

### 4.1. Media Density

As previously stated, all measurements were conducted at a controlled temperature of 25 °C, in order to simulate the processing temperature during the intraoperative isolation of SVF. Given the dearth of existing literature on this topic, we have compared the measured values with the few that have been obtained from other sources. The measured density values for media mixtures differ from the literature by 0.2–0.9% [55,56].

In contrast to the commonly practiced density centrifugation, Drobek et al. used a density meter to determine the density of the cells. They determined the cell number and cell diameter distribution from a 2.5 mL sample volume single-cell suspension and measured the density of the suspension. The measurement permitted the calculation of the cell density [50]. In our study, all media densities determined were close to the density of AD-MSCs described in the literature at $\rho_{DMEM}$ = 1.0525 $\frac{g}{{\mathrm{cm}}^{3}}$ [50] (deviation of media to cell density: (Δρ = 4.5–4.1%)). Thus, negligible radial forces due to rotational shearing processes can be assumed during the measurements performed. The DMEM+G+FCS mixture showed the closest density match to the measured cell density. Unfortunately, the proteins in the fetal calf serum appeared to agglomerate under high shear stress, so it was not suitable as a carrier fluid for long-term shear measurements [57]. The carrier fluid chosen for cell shearing experiments, DMEM+G without fetal calf serum, showed no such agglomerations and had a consistent viscosity over the shear rate range, making it a suitable carrier fluid for the shear force experiments.

### 4.2. Influence of Shear Rate

The viscosity measurements exhibited a low deviation and high repeatability, with a maximum standard deviation of 0.6% observed for water and DMEM/DMEM+G, and a maximum standard deviation of 1.85% observed for DMEM+G+FCS. The mean viscosity of all values under the threshold shear rate for fully laminar tangential flow (${\dot{\gamma }}_{l,tang}$ ≈ 1.5 × 10^4^ s^−1^) results in a 0.13% deviation from Korson’s high precision viscosity measurement of water ($\eta_{H_{2}O}$) = 0.8903 mPas [50], indicating the accuracy of our measurement results.

Since glucose is a rheological modifier, the glucose concentration increases the dynamic viscosity of a mixture [58]. Studies investigating the viscosity of cell media mixtures using a Cannon-Frensje opaque (reverse flow) viscosimeter also showed a change in dynamic viscosity when fetal calf serum was added [55]. These observations are in line with our results. Other studies utilizing a rotational rheometer with a plate–plate configuration showed similar dynamic viscosity values for DMEM and mixtures with fetal calf serum. As the measurement protocol only is described as standard protocol, it can be assumed that only low shear rates were used [56]. No studies on the rheological analysis of DMEM-based cell media mixtures at high shear rates were found, so our viscosity values at high shear rates cannot be compared with the literature values. However, the dynamic viscosity values measured in our study generally showed good agreement with the literature, as evidenced by small differences of less than 5% between the measured viscosity values and the literature values [55,56,58].

### 4.3. Influence of Exposure Time

The viscosity curve exhibited a high degree of consistency over the 10 min observation period, displaying a comparable profile across the entire shear rate range. At an sub-critical shear rate of ${\dot{\gamma \;}}_{radmig,c}$ ≈ 1.0 × 10^4^ s^−1^, the same behavior was observed as at an over-critical shear rate of ${\dot{\gamma \;}}_{radmig,c}$ ≈ 3.0 × 10^4^ s^−1^, indicating a decrease in apparent viscosity due to evaporation rather than radial migration (the initial shear rate for radial migration for DMEM is calculated at (${\dot{\gamma \;}}_{radmig,c}$) ≈ 2.1 × 10^4^ s^−1^) [59]. The data indicate that over a period of 10 min, the apparent viscosity decreased by 8.5%, which can be attributed to the evaporation of the cell media out of the measurement volume. It is recommended that a solvent trap be considered in future experiments to reduce the evaporation of cell media during the course of the experiment [47].

### 4.4. Shear Stress Application Method

Although microfluidic devices offer a wide range of possibilities for applying shear forces to cells and implementing automated real-time monitoring via electrical sensors, the fabrication of microfluidic devices remains a technically challenging and time-consuming process [30]. Recently, Velasco et al. developed a microfluidic impedance platform to measure the response of human umbilical vein endothelium cell monolayers to different fluid shear stress conditions between 1.7 and 5.8 Pa. This platform allowed an automated investigation of sheared cells over 14 h [28]. However, the induced shear stress remains at a physiological level. To reach higher non-physiological shear rates, cannulas are employed as microfluidic channels [29,31]. Barnes et al. employed a cannula as a microfluidic channel to induce shear stress to the cells flowing through. Therefore, he used a 30G standard cannula (D = 0.15 mm) with a length of 1.27 cm. To induce a higher shear stress, the flow rate was raised. The minimum shear rate achieved while maintaining laminar flow in the cannula was between 29 and 640 Pa, but only for a mean transit time of 11.2–0.89 ms [31]. In contrast to microfluidic approaches, the Couette shear device allows the shearing of a cell-loaded liquid with high throughput at high shear rates and exposure time [26,27]. However, special set-ups are used for this, which are expensive and, in contrast to the rheometers used in our approach, are only rarely available.

With the presented method, we were able to effectively demonstrate the application of precise shear stress to cell suspensions at high shear rates. No alterations in the mechanical characteristics of the AD-MSC suspension were detected, including changes in viscosity—such as shear thinning behavior—and no cases of agglomeration were observed in our test approach. Furthermore, several advantages for the induction of shear stress were observed in our experimental set-up in contrast to other shear force application methods. These include the ability to achieve high shear rates over any period of time at low rotational speeds. The cells are not adhered to a monolayer on a plastic 6-well plate or other external device. Instead, the solution is sheared within a high-precision manufactured geometry. This ensures that the cells are subjected to controlled and uniform shear stress. Additionally, the absence of a highly viscous carrier fluid results in lower resuspension requirements and thus less stress on the cells during processing. Consequently, this methodology may be transferable to various cell types.

### 4.5. Shear Response of AD-MSCs

Al-Mofty et al. [23] developed a microfluidic platform for the dissociation of native biological tissue, where computational fluid dynamics (CFD) analyses of different designs showed an average shear stress of 11.5–28.0 Pa in the flow chamber. Scheuermann et al. [24] introduced a grinder device compatible with commercially available standard disposables. This mill-like device features a rotor–stator pair rotated via a benchtop unit. The shearing and milling of murine liver tissue are performed in a 0.1 mm gap at 30–100 rpm within a standard 50 mL conical centrifuge tube, with a shear stress roughly estimated at ~0.85–2.86 Pa. Other systems for the mechanical isolation of the stromal vascular fraction (SVF) commonly employ a combination of enzymatic digestion, centrifugation and shaking, indicating low mechanical forces to the cells similar to Scheuermann and Al-Mofty [22,60]. The studies show the importance of analyzing the impact of high shear rates on cells. In the cases mentioned, it can be assumed that the AD-MSCs are not significantly damaged by the respective shear stresses. The data from our study can also help in the development and analysis of new systems for isolating cells in order to rule out significant damage from the outset.

In terms of shear stress response, previous in vitro studies have primarily characterized the responses of bone marrow-derived mesenchymal stem cells (BM-MSCs) to low to moderate shear stress (typically < 2 Pa), focusing on changes in cell morphology, protein content, gene expression and differentiation potential [61,62,63,64,65]. These studies have shown that moderate shear stress (0.5–2 Pa) can trigger responses in BM-MSCs, such as the upregulation of endothelial cell markers (e.g., PECAM-1, VE-cadherin [61,64]), the downregulation of smooth muscle cell markers (e.g., α-SMA, calponin [61]), increased nitric oxide production and the release, as well as upregulation, of genes such as PTGS2, IER3, EGR1, IGF1 and IGFBP1 and the activation of mechanotransduction pathways (ERK1/2 [66]). The geometry we used in this study allowed a 9–11-fold (18.38–23.05 Pa) higher shear stress compared to the previous studies. Nevertheless, our set-up showed that from a cell biological point of view, the exemplary AD-MSCs used exhibited robust resistance to the applied shear stress periods, as cell viability remained unaffected up to 18.38 Pa for 5 min, the ability to adhere to tissue culture plastic was not impaired by the applied shear rates, and the organization of F-actin within the cytoskeleton retained its physiological phenotype compared to the untreated controls. However, it should be noted that adhesion or changes of the cytoskeleton were examined 24 h after shearing, and a longer-term effect due to prolonged cultivation was not analyzed in detail in this study. However, this should definitely be analyzed in more detail in further studies, as the migration or differentiation potential in particular requires a longer cultivation period. Mechanical destruction of the cells was demonstrated at 23.05 Pa both after 5 and 10 min by a significant increase in the percentage of cell debris. Simmonds et al. found that the threshold between the “normal” and “impaired” cell deformability of red blood cells is between ~80 Pa for short-term exposure (e.g., 1 s) and 38.5 Pa for “infinite” exposure duration [54] (physiologically relevant fluid shear stress for blood cells is 0.1–1 Pa and up to 6 Pa for stenosis [29]). In this context, our results indicate that mesenchymal stem cells derived from adipose tissue have a lower resistance to shear stress compared to red blood cells [54].

In 2019, Curtis et al. demonstrated that MSCs develop into an osteogenic lineage when exposed to a fluid shear stress of 2 Pa at a frequency of 2 Hz in a 2D culture model [67]. Jossen et al. showed that shear stresses of 0.5–2 Pa were sufficient to stimulate the osteogenic differentiation of MSCs in two-dimensional (2D) flow chambers [68]. However, it was observed that intense shear stress appeared to damage the structural integrity of the cells and that a longer treatment duration increased the percentage of cell debris, which agrees with our results. In three-dimensional perfusion bioreactor systems, MSCs are typically exposed to shear stresses in the mPa (millipascal) range, which are 2–5 orders of magnitude lower than in 2D cultures and have been shown to have biological effects on MSCs [61,66]. It has been mentioned that a high shear stress and portal hypertension can impair the adhesion and migration of MSCs, suggesting that excessive shear forces can have deleterious effects [65]. All of these studies clearly show that the cellular responses depend not only on the cell type, but also on factors such as the strength, frequency and duration of the shear stress and whether the stress is intermittent or continuous. It is also important to consider the type of carrier fluid used to apply the shear stress, as well as its composition, such as whether or not it contains a serum. However, some aspects from these studies can be extrapolated to other mesenchymal stem cells; so we hypothesize that AD-MSCs, as another source of mesenchymal stem cells, may show a similar, if not identical, mechanosensitive behavior and differentiation potential when exposed to shear stress [61,62,64,66]. To what extent the migration and differentiation potential of the MSCs used by us is influenced by the low or high shear stress must be the subject of further analyses.

## 5. Conclusions

This study presented a new method for analyzing the impact of high shear stress on cells as a function of selectable time intervals based on a small-angle cone–plate rotational rheometer. In comparison to Couette shearing or microfluidic devices, this method is easy to implement with a rheometer and can be used for various research questions in which the effect of high shear rates on various cells is to be investigated. The results of our initial study with AD-MSCs indicate that the structural integrity of these cells can be affected when exposed to a higher shear stress. Based on our data, fluid shear stresses of 18.38 Pa for up to 10 min exposure time do not harm the viability or structural integrity of cultured AD-MSCs.

With this study, we offer a tool for developers of non-enzymatic dissociation systems to analyze the shear stress resistance of cells and design operating parameters for mechanical dissociation and isolation devices accordingly. This ensures the maximum yield of desired cells during non-enzymatic isolation from donor tissue. Further research is essential to fully understand the specific cellular responses to shear stress and their implications for regenerative medicine. As we only conducted trials using cultured cells, trials should be performed with donor cells to validate our findings. Our results highlight the importance of accurately characterizing shear rates in mechanical isolation systems for regeneratively applied cells to ensure the safety and consistency of both the isolation process and the subsequent application of these cells.

## Figures and Tables

**Table 1:** Dynamic viscosity of cell media mixtures. Mean viscosity values were calculated using measured sub-critical values only. Corrected viscosity following the equation of [48].

Fluid	Mean Viscosity [mPas]	Max SD [± %]	Corrected Viscosity [mPas]
Water	0.8905	0.60	0.8921
DMEM	0.9105	0.56	0.9125
DMEM+G	0.9111	0.57	0.9121
DMEM+G+FCS	0.9572	1.85	0.9642

## Data Availability

The data that support the findings of this study are available from the corresponding author, H.S., upon reasonable request.
